# Comparative Effectiveness of Etanercept and Adalimumab in Patient Reported Outcomes and Injection-Related Tolerability

**DOI:** 10.1371/journal.pone.0149781

**Published:** 2016-03-23

**Authors:** Iris Navarro-Millán, Lisa J. Herrinton, Lang Chen, Leslie Harrold, Liyan Liu, Jeffrey R. Curtis

**Affiliations:** 1 University of Alabama at Birmingham, Birmingham, Alabama, United States of America; 2 Kaiser Permanente, Northern California, San Francisco, California, United States of America; 3 University of Massachusetts, Worcester, Massachusetts, United States of America; Laikon Hospital, GREECE

## Abstract

**Objective:**

To describe patient preferences in selecting specific biologics and compare clinical response using patient reported outcomes (PROs) among patients with rheumatoid arthritis (RA) started on different anti-tumor necrosis factor (TNF) therapies.

**Methods:**

Participants were enrollees in Kaiser Permanente Northern California. Patients with RA who had at least two provider visits and started a new anti-TNF therapy from 10/2010–8/2011, were eligible for participation in this longitudinal study. Using a telephone survey, patient preferences in biologic selection and RAPID3, MDHAQ, and SF-12 scores were collected at baseline and at 6 months. Patient scores rating injection/infusion-site burning and stinging (ISBS) were collected at 6 months.

**Results:**

In all, 267 patients with RA responded to the baseline survey, of whom 57% preferred an injectable biologic, 22% preferred an infused biologic, and 21% had no preference. Motivation for injectable biologics was convenience (92%) and for infusion therapy was dislike or lack of self-efficacy for self-injection (16%). After 6 months of treatment with anti-TNF, 70% of the 177 patients who answered the ISBS question reported ISBS with the last dose; on a scale of 1 (none) to 10 (worst), 41% of these reported a score of 2–5; and 29% reported a score of 6–10. Adalimumab users experienced 3.2 times (95% confidence interval 1.2–8.6) the level of ISBS that etanercept users experienced. There were no significant differences in RAPID3, MDHAQ, or SF-12 scores between etanercept or adalimumab initiators.

**Conclusion:**

Convenience and fear of self-injection were important considerations to patients selecting a biologic drug. Although more convenient, adalimumab associated with more ISBS than did etanercept, and this rate was higher than reported in clinical trials. At 6 months, PROs did not differ between etanercept and adalimumab users.

## Introduction

In general, when medical treatments are being selected, most patients want to be offered choices and the opportunity to give their opinion, even though a sizable proportion may want their physician to make the final decision [1]. For this reason, it is important to understand what matters to patients when choosing a therapeutic. Biologic use among patients with rheumatoid arthritis (RA) and other immune-mediated diseases is growing rapidly [2–5]. However, knowledge of the specific issues that influence patient preferences or decision-making during selection of biologic drugs is limited [6, 7] and most of the studies that have investigated the reasons for treatment choice in RA predominantly addressed physician preferences [8–10]. One study reported that efficacy, safety, and convenience were the most important considerations to patients [6]. Comparative effectiveness studies have shown no significant differences among anti-tumor necrosis factor (TNF) medications in clinical disease activity, measured by the disease activity score in 28 joints (DAS28), clinical disease activity index (CDAI), or serious adverse events [11, 12]. However, when selecting treatments, it is also important that patients have information about the outcomes experienced most directly by other patients, known as patient reported outcomes (PROs). Few head-to-head comparative effectiveness studies of biologics have assessed differences in PROs. Although these studies suggest that patients selecting infused and injected therapies have different definitions of convenience and other differences in priorities [6, 13], further clarification is needed. Therefore, comparative effectiveness studies between any two specific anti-TNFs, purely from the patient and PRO standpoint, are needed.

Furthermore, self-efficacy to administer injections is thought to be important to patients with RA when selecting a biologic agent [14]. It is important to give patients information about the tolerability of injections because patients can experience pain and adverse skin reactions with injectable formulations. The prevalence of these symptoms has been reported to be 15%-20% in clinical trials [15, 16], but information from a survey of patients of 113 community-based rheumatologists indicated a much higher prevalence, about 60%, with 22% of the cohort reporting moderate to severe pain [17]. The objectives of this study, conducted in a cohort of patients with RA enrolled in a large, population-based healthcare system in the U.S., were: 1) to determine factors influencing patients’ decision-making for the selection of specific biologics, focusing on the choice between injectable versus infused agents; 2) to examine the tolerability of subcutaneously administered injections; and 3) to compare PROs between initiators of adalimumab and initiators of etanercept within this cohort.

## Methods

### Patient eligibility and selection

Patients eligible for this study included English-speaking enrollees in Kaiser Permanente Northern California, a large, well-characterized, integrated care organization. Patients with RA were considered preliminarily eligible for the survey if they had at least two provider visits for RA and started a new anti-TNF therapy while enrolled in Kaiser Permanente during the period of October 2010 through August 2011. These individuals were identified within 1 week of initiation using computerized pharmacy information from the electronic medical record and were invited to complete a baseline telephone survey and 6-month follow-up survey. Patients either could have been biologic naïve or could have switched from a previous biologic agent. Permission from the treating rheumatologist and confirmation that the patient had RA were also required. The baseline survey was conducted at the time of initiation of anti-TNF therapy and another survey was conducted 6 months later.

### Surveys

Patients participated in a phone survey. Participants provided a verbal informed consent; a written consent was not feasible given that the survey was conducted over the phone. The assent was recorded in the study database, mainly by the mere fact that we quit the call if they said no. Kaiser Northern California IRB approved this consent procedure and the study. Patient demographics and descriptive information on preferences and factors in selecting an injectable or infused biologic were collected on the baseline survey “S1 Document” for specific details on the survey instrument). Information regarding tolerability of injections and infusions, including pain and injection/infusion-site burning and stinging (ISBS), was collected on the 6-month survey. Patients were asked how much burning and stinging they had with the last biologic dose, rated on a scale from 1 (no pain) to 10 (as bad as it could be). The PROs selected were easy to collect during the survey and were validated to reliably address the data regarding the participant’s disease status. The PRO instruments used in the survey (both at baseline and the 6-month follow-up survey) included the Multidimensional Health Assessment Questionnaire (MDHAQ), the Routine Assessment of Patient Index Data 3 (RAPID3), and the Short Form Health Survey-12 (SF-12). The MDHAQ [18] includes 10 items and queries for four responses: without any difficulty (= 0), with some difficulty (= 1), with much difficulty (= 2), and unable to do (= 3), providing a total score of 0–10 [18, 19]. The RAPID3 [19] measures parameters of disease activity (function, pain, and patient global estimate of status) that are each scored on a scale of 0–10, giving a raw total score of 0–30. Higher scores on the MDHAQ scale and the RAPID3 scale represent worse functional status. The SF-12 physical composite scale (PCS) and mental composite scale (MCS) [20] is a multipurpose short form survey with 12 questions [21]. The questions were combined, scored, and weighted to create two scales that provide glimpses into mental and physical functioning and overall health-related quality of life [22]. The SF-12 uses a scale from 0–100 with a median score of 50 for both the PCS and the MCS components; lower scores suggest worse functional impairment.

### Statistical analysis

Descriptive statistics were used to summarize factors related to preferences for route of administration and for specific biologics, and to summarize the magnitude of ISBS and correlations between ISBS and PROs. This analysis focused on the two biologics used most commonly in the Kaiser Permanente population: adalimumab and etanercept. We used nonparametric statistics to compare the median ISBS scores of adalimumab and etanercept users. Polynomial logistic regression analysis was used to assess factors associated with ISBS after dividing patients into three categories based on their ISBS score: 1–5 (reference group), 6–8, and 9–10. The polynomial logistic regression compared adalimumab with etanercept after adjusting for potential confounders including age, sex, RA disease duration, prior biologic use, and fibromyalgia. The latter two variables were forced into the model based upon clinical interest.

To evaluate the comparative effectiveness of biologics with regard to PROs, we used a multivariate ordinary least square regression to evaluate change in each PRO at 6 months, comparing adalimumab initiators with etanercept initiators and controlling for multiple potential confounders including age, sex, RA disease duration, and prior biologic use. The analysis included all patients who contributed both a baseline and a 6-month survey (per-protocol analysis), irrespective of whether or not they remained on the therapy that they initiated. We also conducted a sensitivity analysis using an intent-to-treat analysis in which the baseline survey PRO was carried forward to the 6-month survey for patients who were lost to follow-up. Patients provided consent to participate, and approval for the study was granted by the Kaiser Foundation Research Institute Institutional Review Board.

## Results

**Fig 1:**
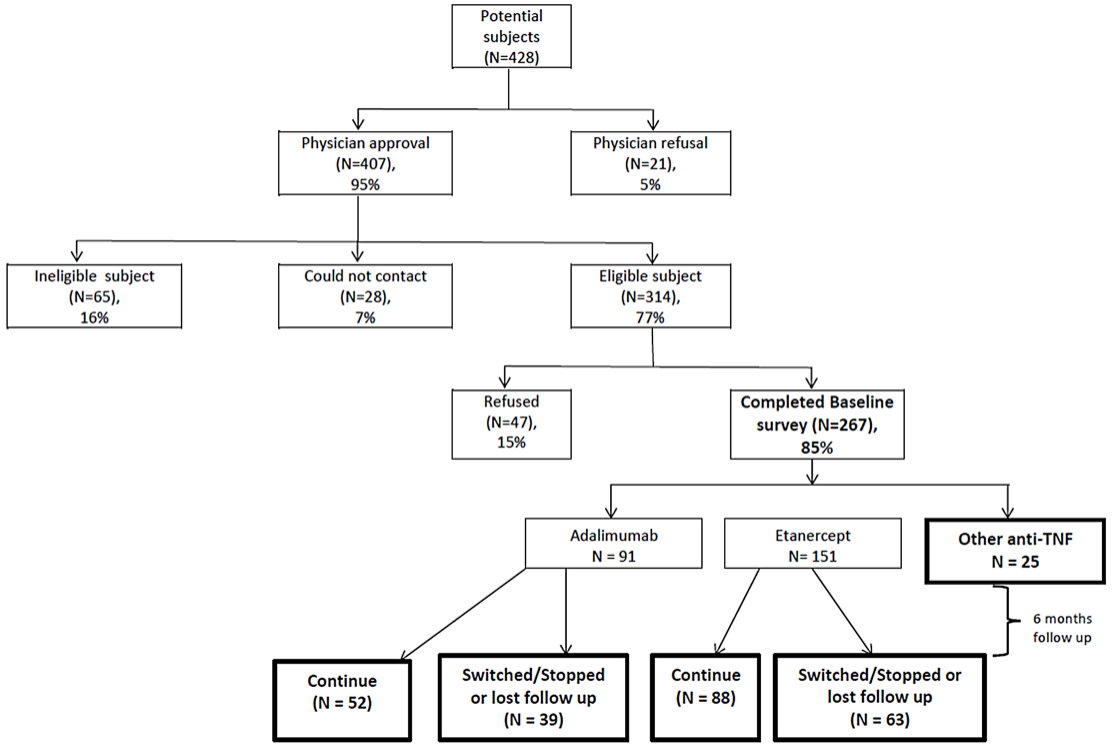
Flow diagram showing patient selection at baseline.

The number of preliminarily eligible patients with RA was 428 (Fig 1), of which 314 received physician approval, could be contacted, and were eligible for the study. Of these, 267 (85%) completed the baseline telephone survey. A minority (N = 25) initiated anti-TNF drugs other than adalimumab or etanercept, and these individuals were not further considered. The remaining 242 patients initiated etanercept (N = 151) or adalimumab (N = 91). Among those patients that initiated etanercept or adalimumab during the baseline survey, 187 (77%) completed the subsequent 6-month follow-up telephone survey (regardless of switching or stopping the initial medication at the 6-month survey).

Mean age of the 242 patients included in the study was 54.4 (SD ± 12.1) years and 73% were females. Further details about patient characteristics are listed in Table 1. At baseline, 56% of patients reported that they participated in the selection of their specific biologic drug. The majority of these (57%) preferred an injectable biologic, 22% preferred an infused biologic, and 21% had no preference. Patients preferring injection biologics were mainly motivated by convenience (92%); those preferring infusion therapy were motivated by dislike of needles or injections overall or lack of self-efficacy for self-injection (16%). A total of 78% of patients reported significant improvement in their RA as the main advantage of taking a biologic drug.

**Table 1:** Etanercept and adalimumab patient characteristics at the time of the baseline survey*.

	Biologic Naïve		Biologic-Experienced	
	ADA (N = 28)	ETA (N = 136)	ADA (N = 63)	ETA (N = 15)
**Demographics and RA-related variables**				
Age (years), mean (SD)	55 (12.3)	53 (11.9)	55 (11.2)	51 (12.7)
Female, %	57	73	84	67
Disease duration (years), mean (SD)	7.3 (12.1)	6.3 (9.0)	11.3 (10.6)	8.6 (7.9)
**Smoking, %**				
Current	7.1	3.7	11.1	13.3
Non-smoker	75.0	88.2	84.1	80.0
Former smoker	17.9	8.1	4.8	6.7
**Race, %**				
Caucasian	46	50	62	60
African American	25	8	5	7
Hispanic	7	21	13	20
Asian	7	2	5	0
Other	15	19	15	13
**Medications**				
Prednisone, %				
None	50.0	57.4	71.4	80.0
1–5 mg	32.1	23.5	22.2	6.7
6–10 mg	10.7	5.2	4.8	13.3
>10 mg	7.1	14.0	1.6	0
**Comorbidities, %**				
Diabetes	7.1	8.8	12.7	13.3
Chronic obstructive pulmonary disease	10.7	15.4	27.0	26.8
Cardiovascular disease	3.6	1.5	1.6	0
Fibromyalgia	10.7	16.9	12.7	13.3
Joint surgery	3.6	0.7	1.6	0

*Baseline survey conducted at the time of initiation of anti-TNF therapy.

ADA, adalimumab; ETA, etanercept.

In all, 177 responders rated ISBS with the last dose of their injectable biologic. The incidence of ISBS for these responders was 70%; 30% of responders reported no ISBS (score = 1 out of a maximum of 10), 41% reported levels between 2 and 5, and 29% reported levels between 6 and 10. Forty-five percent of the patients on injectable biologics listed the level of ISBS or difficulty with needles as a primary inconvenience of using injectable biologics. After 6 months of treatment, 2% of patients initially on an injectable biologic reported that ISBS was the reason they switched to another injectable biologic and 1% reported ISBS as the reason they switched to an infusion biologic. This suggests that despite the fact the ISBS was highly prevalent (70%) it was not a major reason for patients to switch biologics.

**Fig 2:**
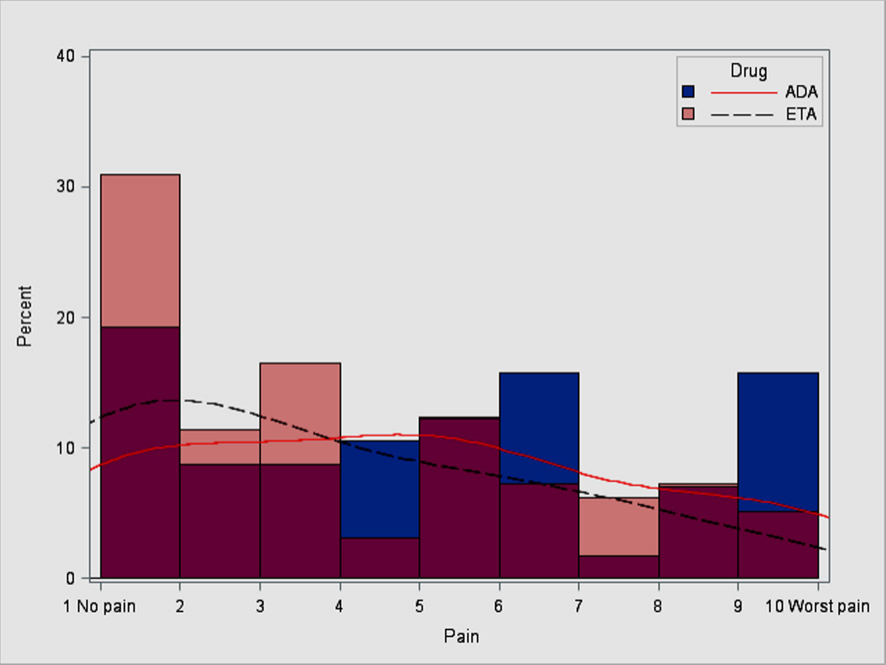
Distribution of ISBS reported for adalimumab and etanercept on the 6-month follow-up survey. Note: The dark pink color represents the overlap between both etanercept and adalimumab.

Of the 187 adalimumab and etanercept initiators who completed the 6-month follow-up survey, 154 (82%) answered the question regarding ISBS. Fig 2 illustrates the level of ISBS for etanercept and adalimumab initiators only. In bivariate analysis, the median (interquartile range (IQR)) level of ISBS for adalimumab and etanercept initiators was 3 (1, 6). The median (IQR) level of ISBS was 5 (2, 6) for adalimumab and 3 (1, 6) for etanercept (p = 0.02). In multivariable adjusted analysis, increasing level of ISBS was associated with RA disease duration (odds ratio [OR] 1.26; 95% confidence interval [CI] 1.04–1.53), younger age (OR 0.69; 95% CI 0.57–0.83), and use of adalimumab compared with etanercept (OR 3.23; 95% CI 1.21–8.62) (Table 2). Level of ISBS was not correlated with change in PROs (data not shown).

**Table 2:** The association of patient and disease factors with ISBS*.

Variable	OR (95% CI)
Adalimumab referent to Etanercept	**3.23 (1.21, 8.62)**
Age, 5-year increment	**0.69 (0.57, 0.83)**
Prior biologic use	0.73 (0.27, 1.95)
RA disease duration (5-year increments)	**1.26 (1.04, 1.53)**
Fibromyalgia	1.02 (0.36, 2.90)

* The analysis used ordinal logistic regression, grouping the response variable as 1–5 (referent); 6–8; or 9–10. 1 = no ISBS and 10 = severe ISBS.

The relationships of infused biologic with improvement in the RAPID3, MDHAQ, and SF-12 are shown in Table 3, which includes only those patients who completed the 6-month survey. For each biologic, each PRO was significantly improved after 6 months of therapy, except the SF-12 PCS in the adalimumab group. For example, the mean MDHAQ improvement exceeded the minimal clinically important difference of 0.22 for both adalimumab (MDHAQ change = 0.8) and etanercept (MDHAQ change = 1.8) [23]. After multivariable adjustment, there were no significant differences between etanercept and adalimumab in any PRO. Age and RA disease duration were marginally associated with improvement in these PROs after multivariable analysis but not to the point of achieving a p < 0.05 (Table 3 footnote). The results of the intent-to-treat analysis, in which the baseline visit data on PRO was carried forward for the missing outcomes at the 6-month follow-up survey, were nearly identical “S1 Table”.

**Table 3:** Crude and adjusted comparison of PROs between adalimumab and etanercept initiators (per-protocol analysis).

Changes in Patient Reported Outcomes (PRO)	Adalimumab Initiators (N = 69)	EtanerceptInitiators (N = 118)
**RAPID3 (0–30 scale)**		
Baseline mean (SD)	15.9 (5.9)	16.6 (5.7)
Crude (mean, SD) improvement at 6 mo compared with baseline^†^	-3.6 (5.5)*	-6.6 (7.1)*
Adjusted mean difference (β, 95% CI) at 6 months	2.2 (-0.32, 4.71)	Referent
**MDHAQ (0–10 scale)**		
Baseline mean (SD)	3.6 (2.0)	3.6 (2.0)
Crude (mean, SD) improvement at 6 mo compared with baseline^†^	-0.8 (1.6)*	-1.3 (1.8)*
Adjusted mean difference (β, 95% CI) at 6 months	0.5 (-0.11, 1.13)	Referent
**SF-12 PCS (0–100 scale)**		
Baseline (mean, SD)	31.2 (10.6)	30.5 (10.2)
Crude (mean, SD) improvement at 6 mo compared with baseline^‡^	1.8 (9.5)***	6.0 (12.6)*
Adjusted mean difference (β, 95% CI) at 6 months	-2.2 (-6.96, 2.65)	Referent
**SF-12 MCS (0–100 scale)**		
Baseline (mean, SD)	46.1 (13.1)	44.3 (12.8)
Crude (mean, SD) improvement at 6 mo compared with baseline^‡^	4.8 (12.4)**	5.9 (13.3)*
Adjusted mean difference (β, 95% CI) at 6 months	0.05 (-4.54, 4.64)	Referent

Includes only patients who completed the baseline and 6-month survey (per-protocol analysis).

Note: the adjusted mean difference at 6 months for each PRO was adjusted for age, gender, RA disease duration, prior biologic, and baseline PRO.

P-values for mean crude changes in PROs

* p = 0.0001

**p = 0.004

***p = 0.16.

RAPID3, Routine Assessment of Patient Index Data 3; MDHAQ, Multidimensional Health Assessment Questionnaire; SF-12 MCS, Short Form Health Survey-12 mental composite scale; SF-12 PCS, Short Form Health Survey-12 physical composite scale; SD, standard deviation; mo = months.

†Negative values for mean MDHAQ and RAPID3 differences at 6 months equals improvement.

‡ Positive values for mean SF-12 PCS/MCS differences at 6 months equals improvement.

## Discussion

To better characterize preferences for biologics and their tolerability and effect on PROs, we obtained and analyzed longitudinal data for a community-based population of patients with RA starting new biologic agents. The study revealed that 56% of patients with RA shared the decision about biologic selection with their physician and that the route of administration (injectable vs. infusion) was important in choosing one biologic over another. The decision about whether to use an infused or injected biologic was largely influenced by the patient’s preference to have the medication administered by a clinician at an infusion clinic or to administer the medication by themselves at home. In addition, the study found no differences in PROs between patients treated with adalimumab and etanercept.

These results were very similar to those observed in a separate community-based study of RA, in which 50% of patients with RA [6] chose injectable anti-TNF [6]. In another study of ankylosing spondylitis, 61% of patients chose injectable anti-TNF because of convenience [13]. The same study revealed that the majority (80%) of the patients chose their biologic as part of a shared decision with their treating physician [13], similar to the present report. Thus, shared decision making between patients and their physicians regarding selection of biologic therapy, as well as the preference for injectable biologics because of convenience, is consistent across populations and rheumatologic diseases.

The findings we report here also are similar to those of a previous study in which the prevalence of ISBS was 59%, compared with our estimate of 70% [17]. Although ISBS was common in our study, it rarely (2%) influenced the patient’s decision to change to a different injectable biologic. A possible explanation is that the overall improvement obtained with the biologic offsets the discomfort of ISBS for the majority of patients; this hypothesis is supported by the lack of correlation between the improvement in PROs and increase in ISBS observed as part of this analysis.

Previous studies demonstrated no difference in ISBS between adalimumab and etanercept, except when the delivery mechanism was taken into account (i.e., pre-filled syringe, syringe, or autoinjector) [17, 24]. The present study did not distinguish between delivery mechanisms used for etanercept and adalimumab, but the association with ISBS was strong, with adalimumab being less well tolerated. Clinicians who do not routinely ask about ISBS may consider doing so because the prevalence of ISBS is high, and for a minority of patients, ISBS may be an important part of their decision in choosing a biologic. There is no clear explanation for differences in ISBS experienced with these biologics, but potential differences in the pH (adalimumab pH = 5.2; etanercept pH = 6.3) or other inactive ingredients could be contributing factors to the differences in ISBS reported in our study [25, 26].

Several studies have suggested differences in treatment response with different anti-TNFs depending on immunogenicity [27–30]; however, these differences generally have not been observed in comparative effectiveness studies that did not make that distinction [11, 12]. The present community-based study confirms that etanercept and adalimumab are similar with regard to effect on PROs. In general, the mean response in each PRO was equal or greater than the minimal clinically important differences in RAPID3 (3.6 units), HAQ (0.22 units), and the PCS and MCS of the SF-36 (5 units), except for the SF-12 PCS for adalimumab [23, 31–33]. This information emphasizes that regardless of the possible decrease in treatment response due to immunogenicity, from the patient perspective (an aspect that has not been studied extensively in comparative effectiveness studies), there is no major difference between the two most commonly used injectable anti-TNFs (adalimumab and etanercept).

Our study has several strengths. Comprehensive outcomes included patient preferences, ISBS, and PROs. Compared with data in past reports; our data has greater generalizability to community-based populations treated by numerous physicians across geographic locations. Furthermore, the sample size (N = 267) was larger than in past studies (N<100) [13, 24]; the response rates to the baseline (85%) and follow-up (77%) surveys were high; and the study was longitudinal, whereas past studies were not [6, 17].

The study also has limitations, including its setting in a single region of the U.S. within a single healthcare system and the inclusion of too few golimumab and certolizumab users for analysis. A minority of patients did not complete the follow-up survey, and we were unable to assess the reason for lack of follow-up, be it an adequate response to treatment or other, although the intent-to-treat analysis indicated that this weakness was unlikely to have a major effect on the study. Another limitation is the short follow-up period between the baseline and follow up survey (6 months). Finally, it is possible that our survey did not include items that could be representative of how the patients felt regarding the use of injectable biologics (e.g., sense of security or aversion to going to the hospital for medication administration).

In conclusion, the selection of biologics for the treatment of RA appears to be a shared decision between patient and physician. Although the prevalence of ISBS was high, especially in adalimumab patients, this did not lead to discontinuation or changes in medication. Etanercept and adalimumab were similar in effectiveness with respect to PROs. Further investigation with longer follow-up data to evaluate patient expectations about effectiveness, safety, and tolerability with regard to medication adherence would be useful for improving long-term outcomes on biologic therapies and to confirm the results of the present study.

## Supporting Information

S1 Document Baseline and Follow up Survey Instrument. (DOCX)

S1 Table Crude and adjusted comparison of patient reported outcomes between adalimumab and etanercept (intent-to-treat analysis). (DOCX)
