# Supplementary material for: Comparative Effectiveness of Etanercept and Adalimumab in Patient Reported Outcomes and Injection-Related Tolerability
Source: PLoS One. 2016 Mar 23;11(3):e0149781. doi: 10.1371/journal.pone.0149781 (PMC4805235; doi:10.1371/journal.pone.0149781)
Supplement: S1 Document — (DOCX) [file pone.0149781.s001.docx]

**S1 Document: Baseline and Follow up Survey Instrument**

**Baseline Survey**

Preferences

| 1. | I’m going to list some drugs. I’d like to know which of these you have discussed with your doctor. Have you and your doctor ever discussed … | | | | | | | | | |  |
| --- | --- | --- | --- | --- | --- | --- | --- | --- | --- | --- | --- |
|  | Etanercept (Enbrel) NO (0)/YES (1) | | | | Abatacept (Orencia) NO (0)/YES (1) | | | | | |  |
|  | Infliximab (Remicade) NO (0)/YES (1) | | | | Anakinra (Kineret) NO (0)/YES (1) | | | | | |  |
|  | Adalimumab (Humira) NO (0)/YES (1) | | | | Rituximab (Rituxin) NO (0)/YES (1) | | | | | |  |
|  | Certolizumab (Cimzia) NO (0)/YES (1) | | | | Tocilizumab (Actemra) NO (0)/YES (1) | | | | | |  |
|  | Golimumab (Simponi) NO (0)/YES (1) | | | |  | | | | | |  |
| 2. | What was your role in selecting your current drug? Would you say… | | | | | | | | | |  |
|  | 1 You made the decision alone. | | | | 4 Your doctor made the decision with your input. | | | | | |  |
|  | 2 You made the decision with your doctor’s help. | | | | 5 Your doctor made the decision alone. | | | | | |  |
|  | 3 You and your doctor made the decision together. | | | | 6 Your doctor made the decision with help from your family.  7 Refused 9 Don’t Know | | | | | |  |
| 3. | Thinking back to your discussions with your doctor, I’d like to understand why [you/your doctor] selected your current drug instead of the other biologic medications that are available. Would you say that your current drug was selected for you because it…. | | | | | | | | | |  |
|  | Is better at treating your arthritis than the other options | | | | | | | | | NO (0)/YES (1) |  |
|  | Is safer | | | | | | | | | NO (0)/YES (1) |  |
|  | Has fewer side effects | | | | | | | | | NO (0)/YES (1) |  |
|  | Is less expensive for you | | | | | | | | | NO (0)/YES (1) |  |
|  | Is less costly to Kaiser | | | | | | | | | NO (0)/YES (1) |  |
|  | Has been around longer, and is more proven | | | | | | | | | NO (0)/YES (1) |  |
|  | 8 Other (Explain) (specify):______________________________________ | | | | | | | | | 7 Refused |  |
|  | 9 Don’t know or can’t remember | | | | | | | | |  |  |
| 4. | If you had a choice and cost was NOT an issue, which one would YOU have chosen? | | | | | | | | | |  |
|  | 1 Self-injection at home every week or two, or | | | | | | | 7 No preference 🡪Skip to G5 | | |  |
|  | 2 Infusion at the clinic every two months  (If response to G4=(2),participant chose infusion,🡪Skip to G4b) | | | | | | | 8 Other (explain)________________🡪Skip to G5  9 Don’t know🡪Skip to G5  777 Refused 🡪Skip to G5 | | |  |
| 4a | (If response to G4=(1),SELF-INJECTION):  Why is that the best choice for you? *Pause then prompt*  Would it be best for you because… | | | | | | | (Text box for comments) | | |  |
|  | It takes less time NO (0)/YES (1) | | | | | | | You don’t have to arrange for childcare  NO (0)/YES (1) | | |  |
|  | You can do it at home NO (0)/YES (1) | | | | | | | You don’t have to take time off work  NO (0)/YES (1) | | |  |
|  | You don’t have to set up appointments NO (0)/YES (1) | | | | | | | 7 Refused | | |  |
|  | You don’t have to worry about transportation and parking  NO (0)/YES (1) | | | | | | | 8 Other (Explain)___________________  9 Don’t know | | |  |
| 4b. | | | (Skip to G5 if G4=(1),participant chose self-injection)  If G4=(2),INFUSION:  Why is that the best choice for you? *Pause then prompt* Would it be best for you because… | | | (Text box for comments) | | | | |  |
|  | | | | You only have to go there once every two months?  NO (0)/YES (1) | | You get other medical care at the same time, such as lab tests or pharmacy pickup?  NO (0)/YES (1) | | | | |  |
|  | | | | You don’t like to give yourself a shot?  NO (0)/YES (1) | | You don’t have to remember to give yourself a shot? NO (0)/YES (1) | | | | |  |
|  | | | | You like to interact with the clinic staff?  NO (0)/YES (1) | | 8 Other (Explain) _______________________ | | | | |  |
|  | | | | 7 Refused | | 9 Don’t know | | | | |  |
| 5. | | If current drug is NOT Infliximab (Remicade) .  On a scale of 1-10 where 1 is “not confident at all” and 10 is “extremely confident,” before you  yourself the first shot of your current drug, how confident did you feel about giving yourself an injection?  Score: ___  1-10  77 Refused 99 Don’t Know | | | | | | | | | |
| 6. | | If current drug IS Infliximab (Remicade).  On a scale of 1 to 10, where 1 is “not at all” and 10 is “very much”, how much do you like going to the clinic?  Score: ___  1-10  77 Refused 99 Don’t Know | | | | | | | | | |
| 7a. | | If previous drug was Infliximab (Remicade).  Previously you used Infliximab (Remicade). Why did you switch to DRUG_NOW?  (Text box for interviewer to record answer)  *Pause and then ask*.  Was it because … | | | | | | | | | |
|  | | The previous drug didn’t help your arthritis enough  NO (0)/YES (1) | | | | | You were worried about safety and side  effects (but didn’t actually have problems  with them)  NO (0)/YES (1) | | | | |
|  | | The previous drug was too expensive for NO (0)/YES (1) | | | | | You had a reaction during or right after  the infusion NO (0)/YES (1) | | | | |
|  | | You had a side effect that required hospitalization NO (0)/YES (1)  *Enter side effect* ___________________________ | | | | | Because you couldn’t get to the clinic as  often as needed? NO (0)/YES (1) | | | | |
|  | | You had a side effect that didn’t require hospitalization  NO (0)/YES (1)  *Enter side effect* ___________________________ | | | | | 7 Refuse | | | | |
|  |  |  |  |  |  |  | 8 Other _______________________________  9 Don’t Know | | | | |
| 7b. | | If previous drug was NOT Infliximab (Remicade):  Why did you switch to current drug?  Was it because … | | | | | | | | | |
|  | | The previous drug didn’t help your arthritis enough NO (0)/YES (1) | | | | | | | You don’t like needles and didn’t like giving  injections? NO (0)/YES (1) | | |
|  | | You had a side effect that required hospitalization  NO (0)/YES (1)  *Enter side effect* ___________________________ | | | | | | | The injection syringe was too hard to use?  NO (0)/YES (1) | | |
|  | | You had a side effect that didn’t require hospitalization NO (0)/YES (1)  *Enter side effect* ___________________________ | | | | | | | You kept forgetting?  NO (0)/YES (1) | | |
|  | | You were too worried about safety and side effects (but didn’t actually have one) NO (0)/YES (1) | | | | | | | It was too expensive, or the insurance didn’t cover  it well enough NO (0)/YES (1) | | |
|  | | You experienced injection site pain, burning, or stinging when you took the shot NO (0)/YES (1) | | | | | | | 7 Refuse  8 Other_______________________________ | | |
|  | | You had to take the medicine more often than was convenient NO (0)/YES (1) | | | | | | | 9 Don’t Know | | |

**Follow up Survey:**

| “On a scale of 1-10, where 1 is ”no problem” and 10 is “as bad as it could be”…..” | | | | | | | | | | | | |
| --- | --- | --- | --- | --- | --- | --- | --- | --- | --- | --- | --- | --- |
| 1. | How much burning and stinging did you have with your last dose of baseline drug? | | | | Score: ____  (1-10) | Refuse  77 | | Don’t Know  99 | | | | |
|  |  | | | | | | | | | | | |
| 2. | Was the amount of injection site burning and stinging you experienced with baseline drug enough to make you want to switch to another self-injected medication? | | | | 0 No | 1 Yes | | 7 Refuse | | 9  Don’t Know | | |
| 3. | Was the amount of injection site burning and stinging enough to make you want to switch to a medication given via infusion? | | | | 0 No | 1 Yes | | 7 Refuse | | 9  Don’t Know | | |
|  | | | | | | | | | | | | |
| “This is a Yes/No question.” | | | | | | | | | | | | |
| 4. | Is the amount of infusion site burning and stinging you experienced with baseline drug enough to make you want to switch to a self-injected medication? | | | | 0 No | | 1 Yes | | 7 Refuse | | 9  Don’t Know | |
|  |  | | | | | | | | | | | |
| 5. | (FOR STOPPERS) What was your role in stopping your baseline drug? | | | | | | | | | | | |
|  | 01 You made the decision alone. | | | 06 Your doctor made the decision with help from your family. | | | | | | | | |
|  | 02 You made the decision with your doctor’s help. | | | 77 Refused | | | | | | | | |
|  | 03 You and your doctor made the decision together. | | | 88 Other________________________________ | | | | | | | | |
|  | 04 Your doctor made the decision with your input. | | | 99 Don’t know | | | | | | | | |
|  | 05 Your doctor made the decision alone. | | |  | | | | | | | | |
|  | | | | | | | | | | | | |
| 6. | | Why did you STOP taking your baseline drug? | | | | | | | | | | |
|  | | 01 You had to take the medicine more often than was convenient | | | | | | | | | | |
|  | | 02 You don’t like needles and didn’t like giving yourself injections | | | | | | | | | | |
|  | | 03 The injection syringe was too hard to use | 10 You were concerned about safety and side effects (but didn’t actually have one) | | | | | | | | | |
|  | | 04 It didn’t help your arthritis enough | 11 Your doctor asked you to stop (text box for comments) | | | | | | | | | |
|  | | 05 It was too expensive, or the insurance didn’t cover it well enough | 77 Refuse | | | | | | | | | |
|  | | 06 You kept forgetting | 88 Other_________________________ | | | | | | | | | |
|  | | 07 You had a side effect that required hospitalization (specify________) | 99 Don’t know | | | | | | | | | |
|  | | 08 You had a side effect that didn’t require hospitalization (specify__________________) | | | | | | | | | | |
|  | | 09 You experienced injection site pain, burning or stinging when you took the shot | | | | | | | | | | |
|  | |  | | | | | | | | | | |
| 7. | | Why did you STOP taking baseline drug (specifically infliximab)? | | | | | | | | | | |
|  | | 01 It didn’t help your arthritis enough | 06 You had a side effect that didn’t require hospitalization specify __________) | | | | | | | | |  |
|  | | 02 Because you couldn’t get to the clinic as often as needed? | 07 You were worried about safety and side effects (but didn’t actually have problems with them) | | | | | | | | |  |
|  | | 03 You had a reaction during or right after the infusion | 08 Your doctor asked you to stop (text box for comments) | | | | | | | | |  |
|  | | 04 It was too expensive, or the insurance didn’t cover it well enough | 77 Refuse | | | | | | | | |  |
|  | | 05 You had a side effect that required hospitalization (specify________) | 88 Other___________________________  99 Don’t Know | | | | | | | | |  |
|  | | | | | | | | | | | | |
| 8. | | (FOR infliximab SWITCHERS) What was your role in SWITCHING your baseline drug to current drug or any other drug in between? | | | | | | | | | | |
|  | | 01 You made the decision alone. | | | 06 Your doctor made the decision with help from your family | | | | | | | |
|  | | 02 You made the decision with your doctor’s help | | | 77 Refuse | | | | | | | |
|  | | 03 You and your doctor made the decision together | | | 88 Other | | | | | | | |
|  | | 04 Your doctor made the decision with your input | | | 99 Don’t know | | | | | | | |
|  | | 05 Your doctor made the decision alone. | | |  | | | | | | | |
| For injectable switchers | | | | | | | | | | | | |
| 9. | | Why did you SWITCH from your baseline drug to current drug or any other drug in between? | | | | | | | | | | |
|  | | 01 You had to take the medicine more often than was convenient | | 08 You had a side effect that didn’t require hospitalization (specify____) | | | | | | | | |
|  | | 02 You don’t like needles and didn’t like giving yourself injections | | 09 You experienced injection site pain, burning or stinging when you took the shot | | | | | | | | |
|  | | 03 The injection syringe was too hard to use | | 10 You were concerned about safety and side effects (but didn’t actually have one) | | | | | | | | |
|  | | 04 It didn’t help your arthritis enough | | 11 Your doctor asked you to switch (text box for comments) | | | | | | | | |
|  | | 05 It was too expensive, or the insurance didn’t cover it well enough | | 77 Refuse | | | | | | | | |
|  | | 06 You kept forgetting | | 88 Other _______________________________ | | | | | | | | |
|  | | 07 You had a side effect that required hospitalization (specify___________________) | | 99 Don’t know | | | | | | | | |
| Only for infusion biologics | | | | | | | | | | | | |
| 10. | Why did you switch from your baseline drug to current drug or any other drug in between? | | | | | | | | | | | |
|  | 01 It didn’t help your arthritis enough | | | 06 You had a side effect that didn’t require hospitalization (specify___________________) | | | | | | | | |
|  | 02 Because you couldn’t get to the clinic as often as needed? | | | 07 You were worried about safety and side effects (but didn’t actually have problems with them) | | | | | | | | |
|  | 03 You had a reaction during or right after the infusion | | | 08 Your Doctor asked you to switch (text box for comments) | | | | | | | | |
|  | 04 It was too expensive, or the insurance didn’t cover it well enough | | | 77 Refuse  88 Other___________________ | | | | | | | | |
|  | 05 You had a side effect that required hospitalization (specify___________) | | | 99 Don’t know | | | | | | | | |

**Supplement 2:**

**Table: Crude and adjusted comparison of patient reported outcomes between adalimumab and etanercept (intent-to-treat analysis)**

|  | **Adalimumab**  **(N = 83)** | **Etanercept**  **(N = 136)** |
| --- | --- | --- |
|  | **All initiators** | **All initiators** |
| **RAPID3,** **(0-30 scale)**  Baseline (mean, SD)  Crude (mean, SD) improvement at 6 months compared to baseline^†^  Adjusted mean difference (β, 95% confidence interval) at 6m | 16.0 (6.1)  -3.0 (5.2)*  1.57 (-0.55, 3.68) | 16.6 (6.2)  -5.7 (7.0)*  Referent |
| MDHAQ  Baseline (mean, SD)  Crude (mean, SD) improvement at 6 months compared to baseline^†^  Adjusted mean difference (β, 95% confidence interval) at 6m | 3.6 (2.0)  -0.7 (1.4)*  0.3 (-0.21, 0.83) | 3.6 (2.0)  -1.2 (1.7)*  Referent |
| **SF-12** **Physical Components Scale**  Baseline (mean, SD)  Crude (mean, SD) improvement at 6 months compared to baseline^‡^  Adjusted mean difference (β, 95% confidence interval) at 6m | 31.0 (10.0)  1.3 (8.3)^***^  -1.68 (-5.31, 1.96) | 30.3 (10.0)  4.8 (11.6)*  Referent |
| **SF-12** **Mental Component Scale**  Baseline (mean, SD)  Crude (mean, SD) improvement at 6 months compared to baseline^‡^  Adjusted mean difference (β, 95% confidence interval) at 6m | 46.0 (13.9)  3.7 (11.0)^**^  0.27 (-3.41, 3.95) | 44.4 (13.2)  4.8 (12.2)*  referent |

**Note: the adjusted mean difference at 6 months for each PRO was adjusted for age, gender, RA disease duration, and baseline patient reported outcome.**

*** P-value = 0.0001; **p-value = 0.004; ***p-value = 0.16**

**RAPID3 = Routine assessment of patient index data 3; MDHAQ = Multidimensional health assessment questionnaire; SF12_mcs = Short form-12 item survey mental composite scale; SF12_pcs = Short form-12 item-pcs survey physical composite scale; SD = standard deviation.**

**†Negative values on mean MDHAQ and RAPID3 differences at 6 months equals improvement. ‡ Positive values on mean SF12-PCS/MCS differences at 6 months equals improvement.**
