# Supplementary material for: Comparative Effectiveness of Etanercept and Adalimumab in Patient Reported Outcomes and Injection-Related Tolerability
Source: PLoS One. 2016 Mar 23;11(3):e0149781. doi: 10.1371/journal.pone.0149781 (PMC4805235; doi:10.1371/journal.pone.0149781)
Supplement: S1 Table — (DOCX) [file pone.0149781.s002.docx]

**S1 Table:**

**Table: Crude and adjusted comparison of patient reported outcomes between adalimumab and etanercept (intent-to-treat analysis)**

| **Changes in Patient Reported Outcomes (PRO)** | **Adalimumab**  **Initiators**  **(N = 69)** | **Etanercept**  **Initiators**  **(N = 118)** |
| --- | --- | --- |
| **RAPID3 (0-30 scale)** |  |  |
| Baseline mean (SD) | 16.0 (6.1) | 16.6 (6.2) |
| Crude (mean, SD) improvement at 6 mo compared with baseline^†^ | -3.0 (5.2)* | -5.7 (7.0)* |
| Adjusted mean difference (β, 95% CI) at 6 months | 1.57 (-0.55, 3.68) | Referent |
| **MDHAQ (0-10 scale)** |  |  |
| Baseline mean (SD) | 3.6 (2.0) | 3.6 (2.0) |
| Crude (mean, SD) improvement at 6 mo compared with baseline^†^ | -0.7 (1.4)* | -1.2 (1.7)* |
| Adjusted mean difference (β, 95% CI) at 6 months | 0.3 (-0.21, 0.83) | Referent |
| **SF-12 PCS (0-100 scale)** |  |  |
| Baseline (mean, SD) | 31.0 (10.0) | 30.3 (10.0) |
| Crude (mean, SD) improvement at 6 mo compared with baseline^‡^ | 1.3 (8.3)^***^ | 4.8 (11.6)* |
| Adjusted mean difference (β, 95% CI) at 6 months | -1.68 (-5.31,1.96) | Referent |
| **SF-12 MCS (0-100 scale)** |  |  |
| Baseline (mean, SD) | 46.0 (13.9) | 44.4 (13.2) |
| Crude (mean, SD) improvement at 6 mo compared with baseline^‡^ | 3.7 (11.0)^**^ | 4.8 (12.2)* |
| Adjusted mean difference (β, 95% CI) at 6 months | 0.27 (-3.41, 3.95) | Referent |

**Note: the adjusted mean difference at 6 months for each PRO was adjusted for age, gender, RA disease duration, and baseline patient reported outcome.**

*** P-value = 0.0001; **p-value = 0.004; ***p-value = 0.16**

**RAPID3 = Routine assessment of patient index data 3; MDHAQ = Multidimensional health assessment questionnaire; SF12_mcs = Short form-12 item survey mental composite scale; SF12_pcs = Short form-12 item-pcs survey physical composite scale; SD = standard deviation.**

**†Negative values on mean MDHAQ and RAPID3 differences at 6 months equals improvement. ‡ Positive values on mean SF12-PCS/MCS differences at 6 months equals improvement.**
